# Polaron Vibronic Progression Shapes the Optical Response of 2D Perovskites

**DOI:** 10.1002/advs.202305182

**Published:** 2023-12-10

**Authors:** Mateusz Dyksik, Dorian Beret, Michal Baranowski, Herman Duim, Sébastien Moyano, Katarzyna Posmyk, Adnen Mlayah, Sampson Adjokatse, Duncan K. Maude, Maria Antonietta Loi, Pascal Puech, Paulina Plochocka

**Affiliations:** ^1^ Department of Experimental Physics Faculty of Fundamental Problems of Technology Wroclaw University of Science and Technology Wroclaw 50370 Poland; ^2^ CEMES‐UPR8011 CNRS University of Toulouse 29 rue Jeanne Marvig Toulouse 31500 France; ^3^ Zernike Institute for Advanced Materials University of Groningen Nijenborgh 4 Groningen 9747 AG The Netherlands; ^4^ Laboratoire National des Champs Magnétiques Intenses EMFL, CNRS UPR 3228 University Toulouse, University Toulouse 3, INSA‐T, University Grenoble Alpes Grenoble and Toulouse France; ^5^ LAAS University of Toulouse CNRS, UPS, 7 Avenue du Colonel Roche Toulouse 31031 France

**Keywords:** 2D perovskites, excitons, polarons, Raman spectroscopy

## Abstract

The optical response of 2D layered perovskites is composed of multiple equally‐spaced spectral features, often interpreted as phonon replicas, separated by an energy Δ ≃ 12 − 40 meV, depending upon the compound. Here the authors show that the characteristic energy spacing, seen in both absorption and emission, is correlated with a substantial scattering response above ≃ 200 cm^−1^ (≃ 25 meV) observed in resonant Raman. This peculiar high‐frequency signal, which dominates both Stokes and anti‐Stokes regions of the scattering spectra, possesses the characteristic spectral fingerprints of polarons. Notably, its spectral position is shifted away from the Rayleigh line, with a tail on the high energy side. The internal structure of the polaron consists of a series of equidistant signals separated by 25–32 cm^−1^ (3–4 meV), depending upon the compound, forming a polaron vibronic progression. The observed progression is characterized by a large Huang‐Rhys factor (*S* > 6) for all of the 2D layered perovskites investigated here, indicative of a strong charge carrier – lattice coupling. The polaron binding energy spans a range ≃ 20–35 meV, which is corroborated by the temperature‐dependent Raman scattering data. The investigation provides a complete understanding of the optical response of 2D layered perovskites via the direct observation of polaron vibronic progression. The understanding of polaronic effects in perovskites is essential, as it directly influences the suitability of these materials for future opto‐electronic applications.

## Graphical abstract

The authors observe the distinct fingerprint of polaron formation in 2D layered perovskites using resonant Raman scattering. The dominating scattering response >200 cm^−1^ is attributed to the polaron vibronic progression with a large Huang‐Rhys factor S>6. Crucially, the observed high‐frequency scattering response is well‐correlated with the characteristic equally separated features in absorption and photoluminescence.

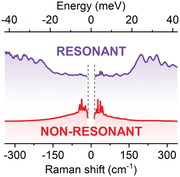

## 1 Introduction

Hybrid perovskites have emerged as a new class of materials with unique electronic properties, which are inherited from both their organic and inorganic constituents. The inorganic framework provides a basis for the semiconducting band structure,^[^ ^1^ ^]^ whilst the organic molecules stabilize the lattice, indirectly controlling the optical properties.^[^ ^2^, ^3^ ^]^ These soft and ionic crystals possess many new and unexpected properties bridging the worlds of classic and organic semiconductors.

A direct consequence of the soft ionic lattice is the significant coupling of charge carriers to the ions in the lattice (i.e., the electron–phonon interaction). Such a strong coupling manifests itself in charge carrier transport and thermalization,^[^ ^4^, ^5^, ^6^ ^]^ dephasing dynamics,^[^ ^7^, ^8^ ^]^ and even structural stabilization.^[^ ^9^ ^]^ Nevertheless, a microscopic description of the electron–phonon coupling is nontrivial, essentially due to the fact that it is mediated by a large anharmonicity and dynamic disorder,^[^ ^5^, ^10^, ^11^ ^]^ which requires the introduction of polarons – a quasiparticle representing charge carriers coupled to the lattice vibrations. Polarons are often regarded as a charge carrier “dressed” in a phonon cloud^[^ ^12^ ^]^ and are characteristic excitations for organic and ionic semiconductors.^[^ ^13^, ^14^, ^15^, ^16^ ^]^ Polarons are widely invoked to understand the low mobilities of charge carriers,^[^ ^17^, ^18^, ^19^ ^]^ the long carrier lifetimes and diffusion lengths^[^ ^20^, ^21^, ^22^ ^]^ in metal halide perovskites.

The most striking consequence of a strong electron–phonon coupling is observed in 2D layered perovskites. Unlike in fully inorganic quantum wells, here the combined effects of quantum confinement and strong dielectric confinement^[^ ^23^, ^24^, ^25^ ^]^ leads to strongly bound excitons,^[^ ^26^, ^27^ ^]^ further complicating the electron–phonon interaction. As a result, the optical response of 2D layered perovskites (absorption and emission)^[^ ^28^, ^29^ ^]^ is composed of multiple equally‐spaced resonances.^[^ ^30^, ^31^, ^32^, ^33^, ^34^ ^]^ Despite the fact that band edge states are derived mainly from the metal and halide orbitals,^[^ ^1^ ^]^ the choice of the organic spacer has a strong influence on the absorption and emission line‐shape,^[^ ^28^, ^29^, ^35^ ^]^ which is a clear manifestation of the hybrid nature of 2D perovskites. A good example of this is provided by the most commonly investigated iodide‐based 2D perovskites, (BA)_2_PbI_4_ (BA ‐ butylammonium, C_4_H_9_NH_3_) and (PEA)_2_PbI_4_ (PEA ‐ phenethylammonium, C_6_H_5_C_2_H_4_NH_3_), in which the energy separation of spectral features in absorption increases from ≃ 15 meV to ≃ 35 meV when a simply aliphatic chains‐based spacer is replaced by a phenyl‐ring‐based molecule.^[^ ^31^, ^32^, ^34^, ^36^ ^]^

A multitude of explanations^[^ ^28^, ^29^, ^30^, ^31^, ^34^, ^37^, ^38^ ^]^ have been offered in an attempt to rationalize the complex optical spectra. In particular, the current poor understanding of emission spectra, where the appearance of multiple peaks has been ascribed to the formation of bi‐excitons,^[^ ^39^ ^]^ in‐gap states^[^ ^40^ ^]^, electron‐phonon coupling^[^ ^41^, ^42^ ^]^ and magnetic dipole transitions,^[^ ^43^ ^]^ limits the progress of applications of 2D perovskites in light emitting devices. Similarly, no consensus exists to explain the complex absorption spectra – magneto‐spectroscopic studies suggest the equally‐spaced features are coupled via the same ground state and the spacing is determined by the energy of the phonon mode to which the exciton couples most strongly.^[^ ^30^, ^32^ ^]^ On the other hand, Thouin et al. invoked polaronic effects, where the equally‐spaced features in spectral response are due to multiple non‐degenerate excitonic transitions, that couple differently to the low‐energy phonon modes of the inorganic lattice to form correlated exciton‐polarons.^[^ ^37^ ^]^

However, neither interpretation is able to quantitatively explain the magnitude of the spacing (≈ 35 meV for (PEA)_2_PbI_4_). Unlike in polar inorganic materials, where the phonon replicas are separated from the free exciton by the energy of the dominating phonon mode in Raman scattering,^[^ ^44^, ^45^ ^]^ the majority of spectral weight in the Raman scattering data of 2D perovskites corresponds to a low‐energy range with frequency ≲ 50 cm^−1^ (≲ 6.2 meV).^[^ ^40^ ^]^ The interpretation involving the formation of a polaronic state also lacks confirmation, as there has been no direct spectroscopic evidence of polarons, in contrast to the case of organic semiconductors.^[^ ^13^, ^46^, ^47^ ^]^ Thus to date, even after thirty or more years investigating 2D perovskites, the detailed origin of such complex excitonic spectra is not thoroughly understood.

In this work, we show that the formation of polaronic states with their characteristic vibronic progression are responsible for complex line shapes observed in 2D perovskites. Crucially, using *resonant* Raman scattering to investigate various 2D layered perovskites, we observe a spectacular shift of the spectral weight to the high‐frequency region above ≃ 200 cm^−1^ (≃ 25 meV), in striking contrast to typical non‐resonant Raman spectra, where the scattering is dominated by the expected low‐frequency optical modes (< 50 cm^−1^). This unusual response, observed in both Stokes and anti‐Stokes regions of the scattering spectrum, possesses all the characteristics of polarons. By directly observing polarons in the resonant Raman scattering we rationalize the origin of the complex absorption and emission spectra of 2D layered perovskites.

## 2 Results and Discussion

**Figure 1:**
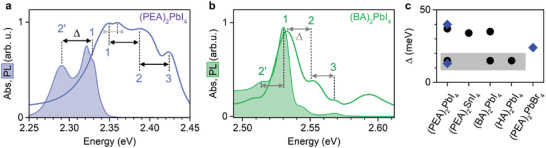
Absorption and PL data of 2D perovskites. Comparison of absorption and PL spectra of a) (PEA)_2_PbI_4_ and b) (BA)_2_PbI_4_. Dashed vertical lines indicate the energy of individual optical transitions. The black (gray) arrows indicate the large Δ > 20 meV (small Δ < 20 meV) energy spacing between the optical transitions. c) Energy difference Δ for several 2D perovskites. The gray region indicates the lower Δ < 20 meV data. Diamonds – literature data after Refs. [30, 31, 32, 34], circles ‐ this work. All data measured at T = 4.2 K.

In **Figure** **1**, we present the optical response of two prototypical perovskites, namely (PEA)_2_PbI_4_ and (BA)_2_PbI_4_, measured at 4.2 K (samples and experimental details can be found in Experimental Section). For both compounds the absorption spectra (solid line) consist of three equally‐spaced distinct transitions labeled 1, 2, and 3 and separated by an energy Δ (indicated by arrows). Associated spectral features, separated by Δ, are also present in photoluminescence (PL) response (shaded curves). This characteristic optical response is observed in many 2D layered perovskites (Figure S1, Supporting Information), as summarized in Figure 1c where we plot measured energetic separation Δ for different compounds.

Although the optical response of 2D layered perovskites is qualitatively similar, there are notable quantitative differences if we compare the Δ values. For PEA‐based perovskites, the reported spacing is much larger (≃35 meV^[^ ^30^, ^31^, ^34^ ^]^) than for the family of the aliphatic‐chain‐based variants (such as BA, HA ‐ hexylammonium, and so on) for which Δ is usually < 20 meV.^[^ ^32^, ^36^ ^]^ At first sight, this suggests that the two families are characterized by a different Δ spacing. However, in high quality optical spectra,^[^ ^31^ ^]^ an additional transition 15 meV above the main absorption peak is also visible in (PEA)_2_PbI_4_ (see e.g., gray arrow in Figure 1a). Thus, we conclude that Δ values <20 meV are not exclusive to the aliphatic‐chain‐based variants. Furthermore, below we will show that the large Δ spacing might actually be expected in both families.^[^ ^34^ ^]^

**Figure 2:**
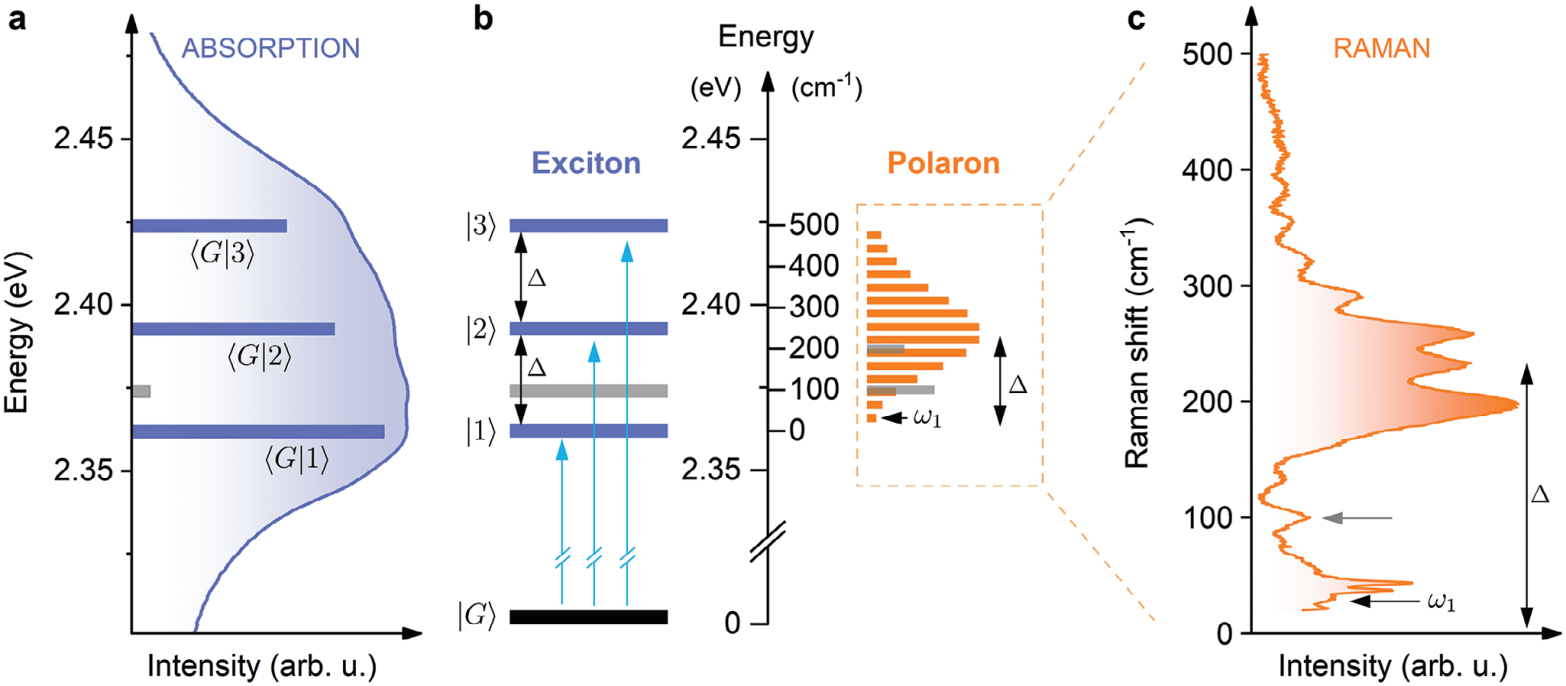
Energy level diagram for excitons and polarons for (PEA)_2_PbI_4_. a) Absorption spectra of (PEA)_2_PbI_4_. Three equally‐spaced (in energy) optical transitions are indicated by horizontal bars. The length of the bars corresponds to the measured transition intensity, determined by fitting with Gaussian functions (Figure S9, Supporting Information). The label 〈*i*|*f*〉 indicates the transition between the initial *i* and final *f* states. |*G*〉 is the ground state (no exciton). The 1s exciton is labeled 〈*G*|1〉. b) The energy diagram of excitons and polarons. The Rayleigh line (0 cm^−1^) is aligned with the energy of the 1s exciton. ω_1_ is the first component in the polaron vibronic progression. Δ denotes the approximate position of the maximum of the Raman response and coincides with the energy difference between the |2〉 and |1〉 absorption signals. Gray bar in the exciton ladder interconnects with the respective Raman signal at ≈ 100 cm^−1^. c) Resonant Raman scattering of (PEA)_2_PbI_4_ (excitation laser: 2.6 eV). Gray arrow indicates the contribution at ≈ 100 cm^−1^. All data measured at T = 77 K.

To elucidate the origin of the complex (Δ separated multi‐peak structure) optical response of 2D layered perovskites, we correlate the spectral features in absorption, with the resonant Raman scattering response. In **Figure** **2** we juxtapose both results for (PEA)_2_PbI_4_ (see Figure S10, Supporting Information for (BA)_2_PbI_4_). The absorption data in Figure 2a consists of equally separated absorption features (Figure S9, Supporting Information), and the horizontal bars indicate the energy of a given transition (length is proportional to oscillator strength). The energy diagram accounting for these optical transitions is presented in Figure 2b, with an energy difference Δ between consecutive absorption features i.e., |*n* + 1〉 and |*n*〉. Such an energy scheme is in full agreement with our previous magneto‐absorption results, where we show that the equidistant absorption features are coupled via a common ground state.^[^ ^30^, ^32^, ^33^ ^]^ Most importantly, a strong response in the high‐frequency range > 200 cm^−1^ is observed in resonant Raman scattering presented in Figure 2c. The spectrum in Figure 2c is unlike any reported to date for 2D layered perovskites – instead of being dominated by low‐frequency modes,^[^ ^40^, ^48^, ^49^, ^50^ ^]^ the maximum intensity is shifted far away from the Rayleigh line to ≃ 200 cm^−1^ (≃ Δ). This new contribution forms an asymmetric comb‐like pattern with a tail on the high energy side. The peaks within the comb are equidistant with a period of ≃ 32 cm^−1^ determined from a FFT analysis (Figure S5, Supporting Information). These properties are simply the well known fingerprint of polarons in organic materials.^[^ ^13^, ^14^ ^]^ More specifically, in the framework of a Holstein polaron,^[^ ^51^, ^52^ ^]^ the observed signal can be ascribed to the formation of a small polaron i.e., a polaron in the strong charge carrier – lattice coupling regime.^[^ ^53^ ^]^

The observed high‐frequency Raman response (Figure 2c) shows similarities with the analytical spectral function in the strong coupling regime $≃\sum_{p=0}^{\infty }\frac{e^{-S}S^{p}}{p!}\delta \left(\omega -p\Delta \omega \right)$ ^[^ ^54^ ^]^ which consists of a series of exactly Δω spaced delta functions at ω_0_, ω_1_,.... The distribution of peak intensities follows a Poisson function with a coupling constant *S*. The peak intensity reaches a maximum value when the level‐index *p* ≃ *S*. Such a Poisson distribution is depicted in the energy diagram presented in Figure 2b by the horizontal orange bars, representing the expected intensity distribution within the Raman response, assuming a large coupling constant *S* ≃ 6 (the precise coupling constant will be determined below). By aligning the Rayleigh line (0 cm^−1^) of the Raman data with the 1s exciton energy in the absorption data (2.363 eV) a direct comparison between both experiments is possible. Clearly, the maximum intensity is shifted by ≃ Δ from 0 cm^−1^ and the center‐of‐mass of the Raman signal (Figure 2c) coincides with the energy level labeled as |2〉 in absorption (see also Figure S10, Supporting Information). In other words, the energy Δ is both the spacing between features in absorption, and the maximum of the resonant Raman response for (PEA)_2_PbI_4_. We emphasize that the successive features in the comb‐like pattern of the resonant Raman describe the polaron ground state and cannot be attributed to the excited states – they are an inherit part of the polaronic response and form a vibronic‐like progression.^[^ ^54^ ^]^

In the resonant Raman response we also observe a contribution at a frequency of ≃ 100 cm^−1^ (gray arrow in Figure 2c), which corresponds to the additional absorption feature 12 meV above the 1s absorption (gray bar in Figure 2a). Thus, for (PEA)_2_PbI_4_ both 12 and ≈35 meV spaced absorption features can be explained by resonant Raman data. However, the relative absorption contribution of the ≈200 cm^−1^ Raman signal (Figure 2b) is masked by the large spectral broadening. We also note the energy scales of the exciton fine structure^[^ ^55^, ^56^ ^]^ (resulting from exchange interaction and crystal field splitting) and self‐trapped excitons^[^ ^57^ ^]^ are order of magnitude different (lower and higher, respectively) than the correction due to polaronic effects.

**Figure 3:**
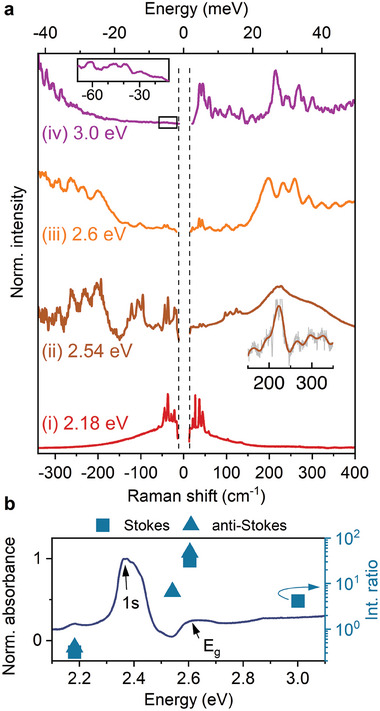
Pump energy dependence on the Raman scattering. a) Stokes and anti‐Stokes (multiplied by exp (− ℏω/*k*_*B*_ *T*) to amplify the signal and normalized) Raman scattering results for different excitation energies. The oscillatory pattern in the Stokes region for the 2.54 eV excitation energy is masked due to hot PL (Figure S7a, Supporting Information). It is, however, clearly visible in the inset after background subtraction. The low‐frequency range of the 3 eV anti‐Stokes spectra (marked with a black rectangle) is enlarged in the inset. b) Linear absorption spectra (solid line) for (PEA)_2_PbI_4_ measured at *T* = 80 K. Data points stand for the integrated intensity ratio of the high frequency (150 − 300 cm^−1^) and low frequency (≲ 50 cm^−1^) Raman response. All data measured at T = 77 K.

At this point, we emphasize the importance of resonant excitation to reveal the polaronic signature in the Raman response of 2D layered perovskites. In **Figure** **3**, we compare the Stokes and anti‐Stokes Raman signals for different excitation conditions: i) non‐resonant (2.18 eV), ii) pre‐resonant with 2s exciton state^[^ ^30^, ^33^, ^58^ ^]^ (2.54 eV), iii) resonant with bandgap^[^ ^27^, ^33^ ^]^ (2.6 eV) and iv) above bandgap (3 eV). One can notice a clear symmetry in the scattering response with respect to the Rayleigh line. The polaronic response is clearly visible under conditions (ii) and (iii), when the excitation energy is close to the bandgap energy. It is symmetric with respect to the Rayleigh line and peaks in intensity around |200| cm^−1^. For energies greater than |200| cm^−1^ the intensity oscillates with a fixed period. The polaronic signal in the Stokes part of (ii) is masked by hot photoluminescence, however, the background corrected spectrum (Figure S7b, Supporting Information) shown in the inset clearly reveals the characteristic intensity modulation. In Figure 3b, we plot the high‐frequency (150 − 300 cm^−1^) to low‐frequency (≲ 50 cm^−1^) intensity ratio for (PEA)_2_PbI_4_ as a function of the excitation energy to quantitatively describe the enhancement of the polaronic response. The intensity ratio data points are superimposed on the linear absorption spectra (solid line). The intensity ratio increases with excitation energy and reaches a local maximum around 2.6 eV, i.e., in the spectral region where the bandgap is located.^[^ ^33^, ^34^ ^]^

It is important to note, that for the above band gap excitation (condition (iv) in Figure 3a), the characteristic high‐frequency features are also present and even dominate the response. However, the FFT analysis of the Stokes region yields a characteristic period of 29.2 ± 0.1 cm^−1^ (Figure S6, Supporting Information), lower than in the case of the (iii) excitation condition. We note that previous studies assigned the high‐frequency Raman signal >200 cm^−1^ to the bending and twisting of the ammonia head (NH_3_) of the organic spacer.^[^ ^28^, ^30^, ^31^ ^]^ Due to the poor signal‐to‐noise ratio with 3 eV excitation, we cannot rule out that the NH_3_ modes also contribute to the observed scattering response. Further studies, both experimental and theoretical, are needed to understand the reduced period for the above bandgap excitation condition, as well as the mutual dependence between the modes of the organic and inorganic sublattices.

A similar difference in the Raman response for resonant and non‐resonant excitation is also observed for other 2D layered perovskites. When Pb is substituted by Sn i.e. (PEA)_2_SnI_4_ the resonant Raman scattering peaks at ≈240 cm^−1^ (Figure S3, Supporting Information), which is consistent with the Δ spacing for this compound (Figure S1, Supporting Information). For (BA)_2_PbI_4_ the resonant Raman spectrum (Figure S4, Supporting Information) reveals the strongest signal at 116 cm^−1^ to which the exciton couples most strongly.^[^ ^59^ ^]^ In addition, we also observe a typical polaron vibronic progression at higher frequencies > 200 cm^−1^, however, the intensity of this scattering is much smaller than the dominant mode at 116 cm^−1^.

We emphasize on the observed correlation between the resonant Raman scattering response and absorption/PL data. We note the difference between the resonant scattering response of (PEA)_2_PbI_4_ and (BA)_2_PbI_4_ – the spectral weight is shifted from ≈ 250 cm^−1^ (≈ 31 meV) to 116 cm^−1^ (≈ 14 meV), respectively (Figure S11, Supporting Information). These energies directly correspond to the characteristic Δ spacing between consecutive signals in absorption/PL observed for both these compounds.

In all investigated samples a general trend is visible – the polaronic signal is evident when the excitation is near the bandgap energy. This suggests that electronic excitation plays an important role in the generation of the unusual Raman response. It is visible only if the excitation is resonant with the electronic transition of the system, as expected if there is a strong coupling between electronic and lattice excitations.^[^ ^37^ ^]^

**Figure 4:**
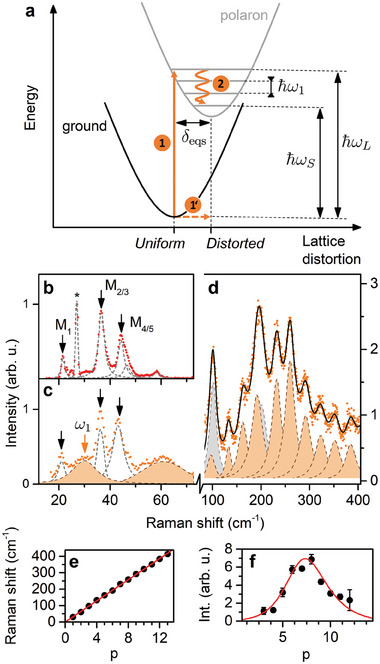
Determination of the Huang‐Rhys factor. a) Configuration coordinate diagram illustrating the potentials as a function of lattice distortion for ground and polaron states. The potentials are displaced by the lattice displacement parameter *δ*_eqs_. ℏω_*L*_, ℏω_*S*_, and ℏω_1_ are the excitation energy, energy of the scattered photon, and phonon energy, respectively. Orange arrows with 1 − 2 labels depict the Stokes resonant Raman scattering process. The low‐frequency portion of the scattering spectra for (b) non‐resonant (2.18 eV) and (c) resonant (2.6 eV) excitation conditions. Black arrows indicate the main optical modes of the inorganic sublattice. The orange shaded peaks spanning through panels (c, d) constitute the polaron vibronic progression, with the first element ω_1_ marked with an orange arrow. e) The energy of consecutive peaks in the progression. The solid red line is a linear fit. f) The intensity of consecutive signals building up the polaronic response. The line stands for fit with Equation (1). The gray shaded peaks in (d) correspond to the ≈ 100 cm^−1^ signal and its overtones.

The striking contrast between the resonant and non‐resonant scattering response suggests a scalable reorganization of the position of the atoms upon photo‐excitation,^[^ ^60^, ^61^, ^62^ ^]^ leading to local lattice deformation and polaron creation.^[^ ^10^, ^15^, ^63^, ^64^ ^]^ For resonant Raman scattering such a process is depicted in **Figure** **4**a in the form of a configurational coordinate diagram. Upon resonant excitation two processes follow; (process 1, Figure 4a) the charge carrier is excited and (process 1') the lattice undergoes a dynamic distortion which lowers the energy of the system.^[^ ^65^ ^]^ Thus the excited charge carrier is surrounded by a phonon cloud, forming a polaronic state. The system subsequently emits *p* optical phonons (total energy loss *p*ℏω_1_, process 2) followed by a “scattered” photon of energy ℏω_*S*_ (ℏω_*S*_ = ℏω_*L*_‐*p* ℏω_1_). The Raman scattering cross‐section at finite temperature *T* involving the successive emission of *p* optical phonons is given by [66, 67],

(1) $${\left|W\right|}^{2}∼{\left|\frac{{\left(\frac{\bar{n}+1}{\bar{n}}\right)}^{\frac{p}{2}}e^{-S(2\bar{n}+1)}I_{p}\left(2S\sqrt{\bar{n}\left(\bar{n}+1\right)}\right)}{E_{g}+p\hbar \omega_{1}-\hbar \omega_{L}+i\gamma }\right|}^{2}$$

where $\bar{n}={\left(e^{\frac{\hbar \omega_{1}}{k_{B}T}}-1\right)}^{-1}$ is the occupation factor, *k*_*B*_ is the Boltzman constant, *I*_*p*_ is the *p*th order modified Bessel function, γ is the broadening parameter, *E*_*g*_ is the bandgap and *S* is the Huang‐Rhys factor.

To determine the Huang‐Rhys factor *S* characterizing the strength of the charge carrier–phonon coupling, we begin by analysing the intensity of the *p*th consecutive peaks in the progression (*p* > 0, *p* = 0 corresponds to the Rayleigh (elastic) scattering process). In Figure 4b–c, we juxtapose the low‐frequency scattering response of (PEA)_2_PbI_4_ for non‐resonant and resonant excitation conditions. The low‐frequency optical modes labeled M_*X*_ (after Thouin et al.^[^ ^37^ ^]^) correspond to the vibrations of the inorganic cage.^[^ ^40^, ^48^, ^49^, ^50^ ^]^ We highlight the remarkable agreement with exquisitely beautiful impulsive stimulated Raman measurements^[^ ^37^ ^]^ where the low‐frequency optical modes are observed in the FFT spectrum of the oscillatory component of the time‐resolved differential transmission spectrum. Alongside these modes in the resonant spectrum (Figure 4c), we observe an additional contribution marked with an orange arrow attributed to the first component of the polaron vibronic progression ω_1_ at 30 ± 1.6 cm^−1^ which is followed by ω_2_ (≈ 60 cm^−1^), ω_3_ (≈90 cm^−1^) and so on. The cumulative curve for the high‐frequency components is presented in Figure 4d. The additional signals at ≈ 100 and its overtone at 200 cm^−1^ are rationalized from the comparison between the resonant Raman spectra of (PEA)_2_PbI_4_ and (BA)_2_PbI_4_ (Figure S11, Supporting Information). This signal corresponds to the lower limit of Δ <20 meV as discussed above.

In Figure 4e, we plot the frequencies of the given components of the polaron vibronic progression as a function of the number of phonons emitted *p*, for values of *p* as large as 13. We find a linear dependence with a slope of 31.7 ± 0.3 cm^−1^, in agreement with the period determined from FFT analysis (Figure S5, Supporting Information). The intensity of the *p*th component is summarized in Figure 4f. By least‐square fitting (input parameters summarized in Table S1, Supporting Information) the data points in Figure 4f with Equation (1), we determine the Huang‐Rhys‐factor *S* = 8.6 ± 1.3 (for the ℏω_1_ mode). We note that previously reported values for (PEA)_2_PbI_4_ (non‐resonant Raman scattering) equal to *S* = 0.44 − 2.15 and were reported for phonon modes of energy > 8 meV.^[^ ^34^ ^]^ The much larger value determined in this work is directly related to the substantial lattice deformation upon resonant excitation^[^ ^60^, ^61^, ^62^ ^]^ described by the dimensionless lattice displacement *δ*_eqs_ parameter (see Figure 4a). The identity $S\approx \delta_{\mathrm{eqs}}^{2}$ ^[^ ^68^ ^]^ directly connects the Huang‐Rhys factor with the variation of the equilibrium positions of the lattice *δ*_eqs_.^[^ ^66^ ^]^ In other words, the resonant excitation leads to a larger lattice displacement *δ*_eqs_, and a polaron formation, resulting in a large Huang‐Rhys‐factor.

By performing similar investigations for different 2D layered perovskites, namely (PEA)_2_SnI_4_ and (BA)_2_PbI_4_ we find that our observations are universal to this class of materials. Under resonant excitation, we consistently observe a high‐frequency comb‐like signal at energies > 200 cm^−1^. For (PEA)_2_SnI_4_ and (BA)_2_PbI_4_ (Figures S3 and S4, Supporting Information, respectively) the polaron vibronic progression begins with ω_1_ at a frequency of 27.7 ± 0.3 and 25.2 ± 0.2 cm^−1^, respectively, and the respective Huang‐Rhys factors are *S* = 7.5 ± 1.1 and *S* = 6.3 ± 1.1. Having both *S* and ℏω_1_ we estimate the lower bound for polaron binding energy^[^ ^64^, ^69^ ^]^ *E*_*p*_ = *S*ℏω_1_ equal to 33.8 meV for (PEA)_2_PbI_4_. The determined parameters are summarized in **Table** **1**. We note the estimated *E*_*p*_ are of the same order as recently determined from ps‐scale time‐resolved photoluminescence.^[^ ^70^ ^]^

**Table 1:** The properties of the polaron vibronic progression. From left: first component of polaron vibronic progression ω_1_, Huang‐Rhys factor *S* and polaron binding energy *E*_*p*_.

	ω_1_ [cm^−1^]	*S*	*E*_*p*_ [meV]
(PEA)_2_PbI_4_	31.7 (0.3)	8.6 (1.3)	33.8 (4.3)
(PEA)_2_SnI_4_	27.7 (0.3)	7.5 (1.1)	25.8 (3.9)
(BA)_2_PbI_4_	25.2 (0.2)	6.3 (1.1)	19.7 (3.4)

**Figure 5:**
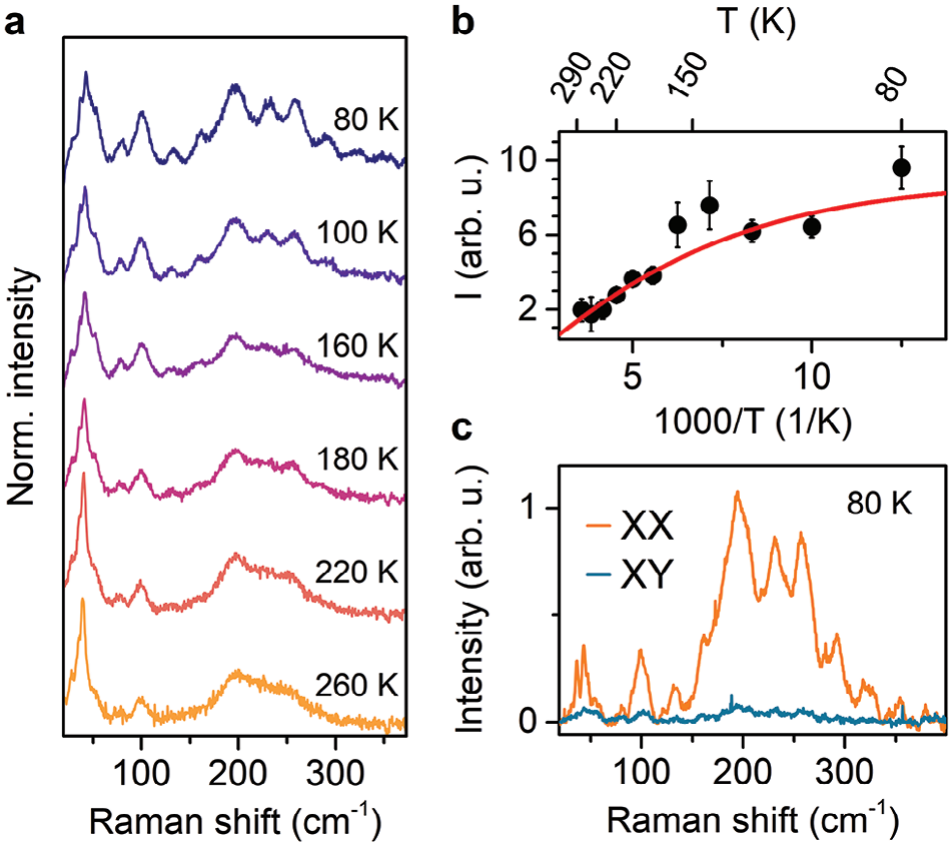
Temperature and polarization properties of polaronic response in (PEA)_2_PbI_4_. a) Temperature‐dependent resonant Raman scattering spectra (exc. 2.60 eV). b) Integrated intensity of the polaronic response (in 150 – 350 cm^−1^ range). The solid line stands for fit with the Arrhenius formula. c) Polarization resolved Raman scattering of polaronic response.

To corroborate the determined *E*_*p*_ with the polaron dissociation, we have performed resonant Raman scattering at different temperatures as presented in **Figure** **5**a. With increasing temperature the spectral weight changes; the low‐frequency modes dominate the spectrum at elevated temperatures where the polaron vibronic progression is less visible. In order to estimate the thermal activation energy of the high‐frequency response, we calculate the integrated intensity *I* of the high‐frequency signal at given temperature *T*. This temperature dependence, plotted in Figure 5b, is modeled with $I\left(T\right)=I_{0}/\left(1+Ce^{-\frac{E_{a}}{k_{B}T}}\right)$, where the *E*_*a*_ is the thermal activation energy, *C* the amplitude parameter and *k*_*B*_ the Boltzman constant. The fitting procedure yields *E*_*a*_ = 22.7 ± 10.9 meV, a value reasonably close to the polaron binding energy *E*_*p*_ for (PEA)_2_PbI_4_.

In Figure 5c, we present the polarization‐resolved Raman scattering spectra. We observe a typical response for the co‐polarized configuration (XX) and a vanishing scattering for the cross‐polarized (XY) configuration.^[^ ^71^ ^]^ Given that the investigated samples are polycrystalline films, the observed Raman modes, together with the polaronic response, are clearly highly symmetric. Both low‐frequency Raman modes and high‐frequency polaronic signal behave in the same manner, which further confirms that the polaron vibronic progression is a result of strong coupling between the charge carriers and lattice vibrations where the lattice thermalization is mostly driven by the inorganic modes shaping the high‐frequency response.

## 3 Conclusions

To summarize, we have observed the distinct fingerprint of polaron formation in 2D layered perovskites using resonant Raman scattering. The strong scattering response >200 cm^−1^ is attributed to the polaron vibronic progression with a large Huang‐Rhys factor *S*. The large Huang‐Rhys factors *S* > 6 determined here indicate the strong charge carrier – lattice coupling regime. Crucially, the observed high‐frequency scattering response is well‐correlated with the Δ‐energy separated features in absorption and PL. Thus, our observations unambiguously explain the optical response of many 2D layered perovskites, where a rich multi‐feature optical response is observed, and can be correlated to, and explained by polaronic effects. The relatively straightforward means used to observe polarons, presented in this work, provides new possibilities to tailor the lattice relaxation pathways for the optimization of opto‐electronics based on 2D layered perovskites. Perhaps more importantly, it also opens the field of *polaronics* in these materials.

## 4 Experimental Section

### Optical Spectroscopy Setup

Basic optical characterization was measured with a fiber‐based setup. For transmission and reflectivity a tungsten halogen lamp was used as a broad‐band white light source. The source light was sent to the sample using an optical fiber (100 µm core) and focused on the sample with a lens (excitation spot ≃ 1 mm^2^). The transmitted/reflected light was collected using a second fiber (400 µm core). The collected light was dispersed and detected using a monochromator equipped with a diffraction grating and a nitrogen‐cooled CCD camera. The sample was installed in a variable‐temperature helium cryostat. For photoluminescence measurements the 407 nm laser was used as an excitation source and the emitted signal was acquired in the same geometry.

### Raman Scattering Experiments

The multi‐wavelength Raman spectra were carried out using a triple monochromator Raman spectrometer T64000 from Jobin‐Yvon Horiba company associated with Argon and Krypton gas lasers. It was possible to approach the Rayleigh line to within 20 cm^−1^ on both the Stokes and anti‐Stokes sides. The samples were sealed in a quartz cell with an inert atmosphere before cooling to avoid contact with moisture. The quartz cell was thermally connected to the cold finger using a thermal paste. The PI micro‐cryostat, able to monitor the sample temperature, was purged with N_2_ (without heating) and then, the sample was cooled to low temperature (≈ 77 K). In all cases, an objective with ×40 magnification and a laser power of typically less than 1 mW were used. The sample was systematically inspected optically confirming the absence of degradation.

### Sample Synthesis Details

Glass substrates were ultrasonically cleaned sequentially using a detergent solution, deionized water, acetone, and isopropanol. Subsequently, the substrates were dried in an oven *T* = 140 °C for ⩾10 min before treatment with ultraviolet ozone for 20 min. Immediately after cleaning, the substrates were placed in a nitrogen‐filled glove‐box for film deposition.

A stoichiometric precursor solution was used, prepared by dissolving PEAI (98.0% TCI) and PbI_2_ or SnI_2_ at a molar ratio of 2:1 in a mixed solvent of DMF and DMSO (4:1 volume ratio, 0.5M concentration). In order to homogenize the solutions, they were stirred for at least 3 h at room temperature prior to deposition. A spin‐coating process with antisolvent treatment was used to deposit the precursor solution onto the cleaned substrates. A rotation speed of 2000 rpm was used for the first 10 s of the spin coating process. The speed was then accelerated to 8000 rpm for the remaining 30 s. 5 s prior to the end of the spin coating cycle the antisolvent (chlorobenzene) was added to the substrate. The films were immediately annealed at 100°C in a nitrogen atmosphere for 10 min.

## Conflict of Interest

The authors declare no conflict of interest.

## Supporting information

Supporting Information

## Data Availability

The data that support the findings of this study are available in the supplementary material of this article.
