# Supplementary material for: Polaron Vibronic Progression Shapes the Optical Response of 2D Perovskites
Source: Adv Sci (Weinh). 2023 Dec 10;11(7):2305182. doi: 10.1002/advs.202305182 (PMC10870061; doi:10.1002/advs.202305182)
Supplement: Supplementary file 1 — Supporting Information [file ADVS-11-2305182-s001.pdf]

ADVANCED SCIENCE
Open Access

Supporting Information

for *Adv. Sci.*, DOI 10.1002/advs.202305182

Polaron Vibronic Progression Shapes the Optical Response of 2D Perovskites

Mateusz Dyksik, Dorian Beret, Michal Baranowski, Herman Duim, Sébastien Moyano, Katarzyna Posmyk, Adnen Mlayah, Sampson Adjokatse, Duncan K. Maude, Maria Antonietta Loi, Pascal Puech and Paulina Plochocka**

Supplementary Information
Polaron Vibronic Progression Shapes the Optical Response of 2D Perovskites

Mateusz Dyksik,[1,*] Dorian Beret,[2] Michal Baranowski,[1] Herman Duim,[3] Sébastien Moyano,[2] Katarzyna Posmyk,[1,4] Adnen Mlayah,[5] Sampson Adjokatse,[3] Duncan K. Maude,[4] Maria Antonietta Loi,[3] Pascal Puech,[2] and Paulina Plochocka[4,1]

[1]*Department of Experimental Physics, Faculty of Fundamental Problems of Technology, Wroclaw University of Science and Technology, Wroclaw, Poland*

[2]*CEMES-UPR8011, CNRS, University of Toulouse, 29 rue Jeanne Marvig, 31500 Toulouse, France*

[3]*Zernike Institute for Advanced Materials, University of Groningen, Nijenborgh 4, 9747 AG Groningen, The Netherlands*

[4]*Laboratoire National des Champs Magnétiques Intenses, EMFL, CNRS UPR 3228, University Grenoble Alpes, University Toulouse, University Toulouse 3, INSA-T, Grenoble and Toulouse, France*

[5]*LAAS, University of Toulouse, CNRS, UPS, 7 Avenue du Colonel Roche, Toulouse, 31031, France*

(Dated: November 23, 2023)

SUPPLEMENTARY FIGURES

FIG. S1. **OPTICAL RESPONSE OF PEA-BASED 2D PEROVSKITES**

Transmission (dotted line) and PL (shaded curves) for **a** $(PEA)_2SnI_4$ **b** $(PEA)_2PbI_4$ and **c** $(PEA)_2PbBr_4$. The vertical lines indicate energy positions of equidistant absorption (emission) peaks labeled 0, 1, 2. The dominant emission in (b) is due to the localized excitons[1, 2].

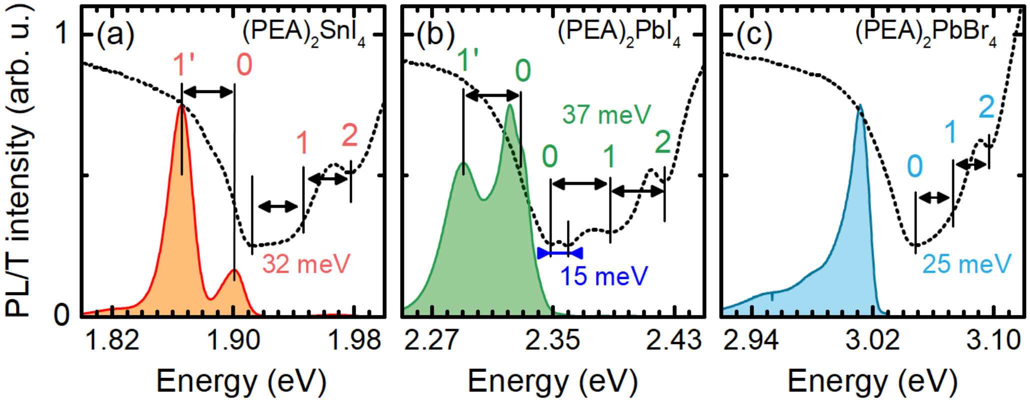

FIG. S2. **RAMAN SCATTERING DATA TREATMENT**

In order to determine the mode intensity from the Raman scattering data the following procedure is used: (i) the background is modeled on the basis of the 280 K spectrum. This spectrum has a vanishing and strongly broadened polaron progression and the low energy modes are of low intensity there. The simulated background is presented as a dashed line in Fig. S2. (ii) The generated background is subtracted from all the data yielding a spectra presented in Figs. 2, 4, 5

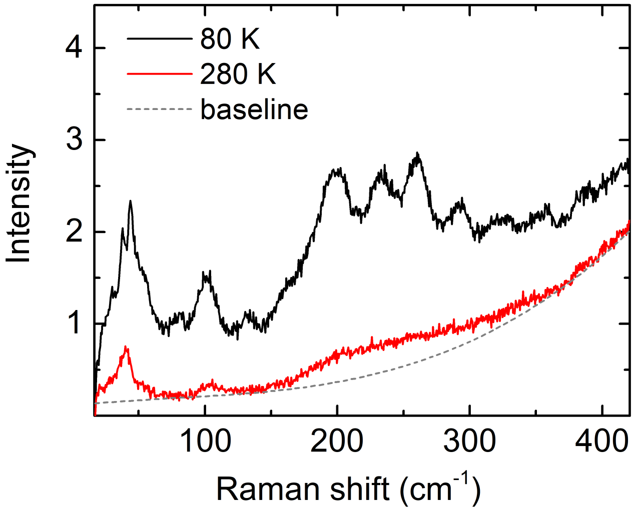

.

FIG. S3. **RAMAN SCATTERING OF $(PEA)_2SnI_4$**

a Non-resonant (red) and resonant (green) Raman scattering. Clearly the resonant response shows a shift of spectral weight towards higher frequencies $> 200\,\mathrm{cm}^{-1}$. To better visualize the intensity modulation in resonant scattering in **b** we show the background subtracted spectrum. **c** The FFT spectrum of the background subtracted signal in panel **b**. The red curve is a Lorentz fit yielding the period of 27.5 ± 0.2 cm^{-1}. **d** The intensity of the consecutive signals in the polaron vibronic progression. Solid line stands for Poisson distribution (parameters in Tab. I in main text). **e** The frequency of signals in the progression vs. its ordinal number. Solid line is a linear fit yielding $\omega_1 = 27.7 \pm 0.3$ cm^{-1}.

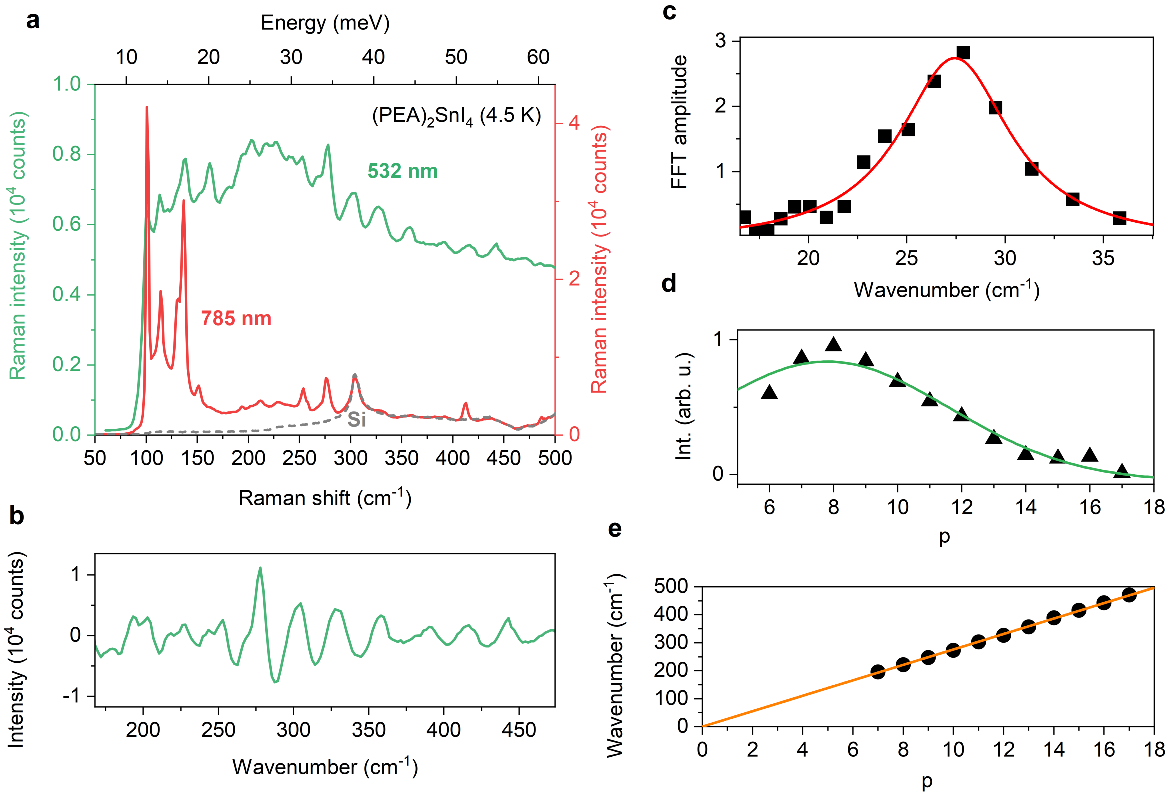

FIG. S4. **RAMAN SCATTERING OF $(BA)_2PbI_4$**

a Stokes and anti-stokes Raman scattering for different excitation energies. **b** High-frequency scattering response indicated by dashed rectangle in panel a. Similar to $(PEA)_2PbI_4$ (discussed in the main text) this response consist of equidistant signals (green peaks) understood as the polaron vibronic progression. The gray-shaded contribution at $\sim$ 220 cm^{-1} is the overtone of the $\sim$ 110 cm^{-1} signal. **c** The intensity and **d** frequency of the signals building up the polaron vibronic progression. The solid line in **c** stands for a fit with a Poisson distribution and the fitting results are summarized in Tab. I in the main text. In **d** the linear fit yields the $\omega_1 = 25.2 \pm 0.2$ cm^{-1}.

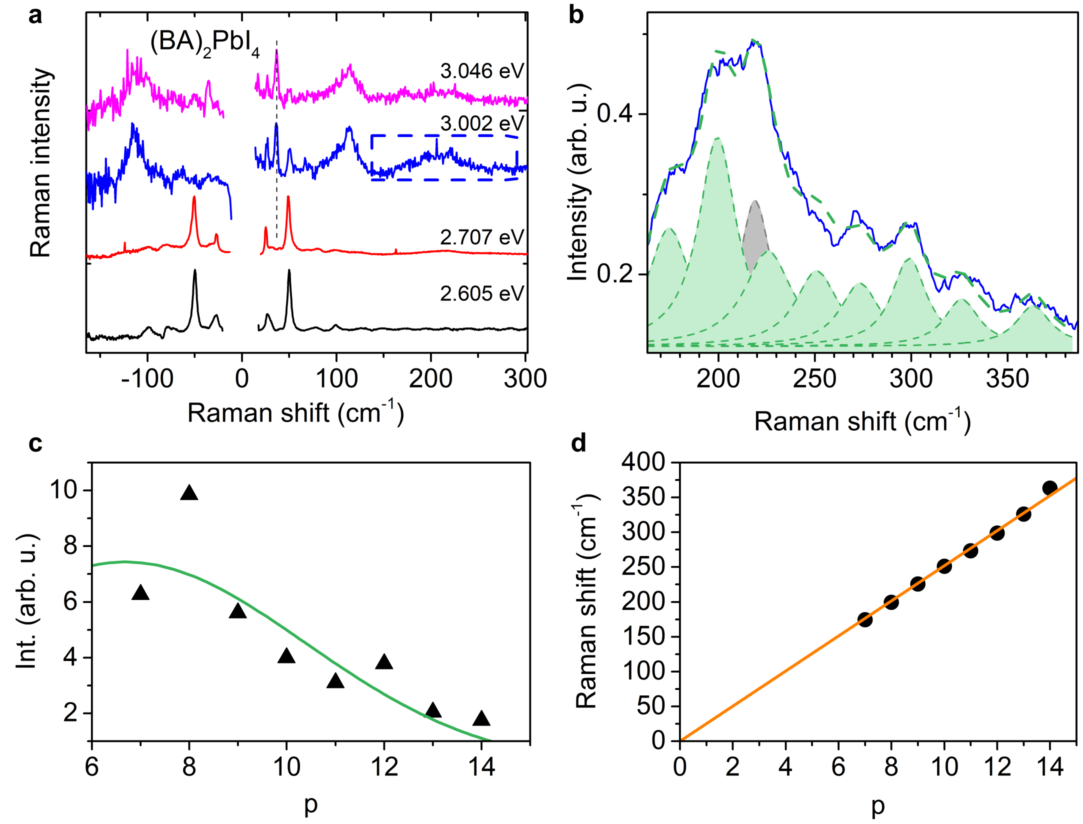

FIG. S5. **FFT OF RESONANT RAMAN SCATTERING OF $(PEA)_2PbI_4$**

a The periodic component of the resonant Raman scattering spectrum for $(PEA)_2PbI_4$ measured with 2.6 eV excitation (Fig. 3 in main text) obtained by subtracting slowly varying background. The background is generated by a Savitzky-Golay filter with a 60 point smoothing windows (for example see Fig. S7). **b** The FFT spectrum of the oscillatory pattern in panel a. The solid red line is a Gaussian fit yielding a period of $31.6 \pm 0.1\,\mathrm{cm}^{-1}$.

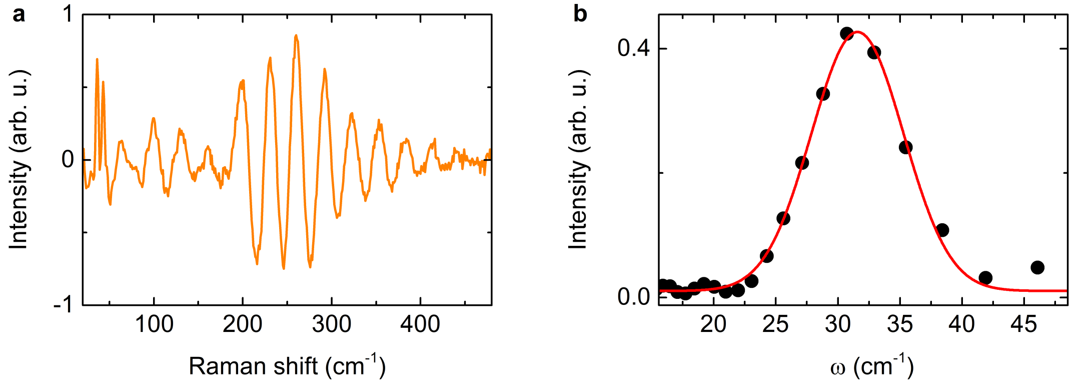

FIG. S6. **FFT OF RESONANT AND ABOVE BAND GAP RAMAN SCATTERING OF $(PEA)_2PbI_4$**

a The periodic component of the Raman scattering data for $(PEA)_2PbI_4$ measured with 2.6 eV and 3 eV excitations (Fig. 3 in main text) obtained by subtracting slowly varying background. The background is generated by a Savitzky-Golay filter with a 60 point smoothing windows (for example see Fig. S7) **b** The FFT spectrum of the oscillatory pattern in panel a. The solid lines are a Gaussian fits yielding a period of $31.6 \pm 0.1\,\mathrm{cm}^{-1}$ and $29.2 \pm 0.1\,\mathrm{cm}^{-1}$ for 2.6 and 3 eV excitation conditions, respectively.

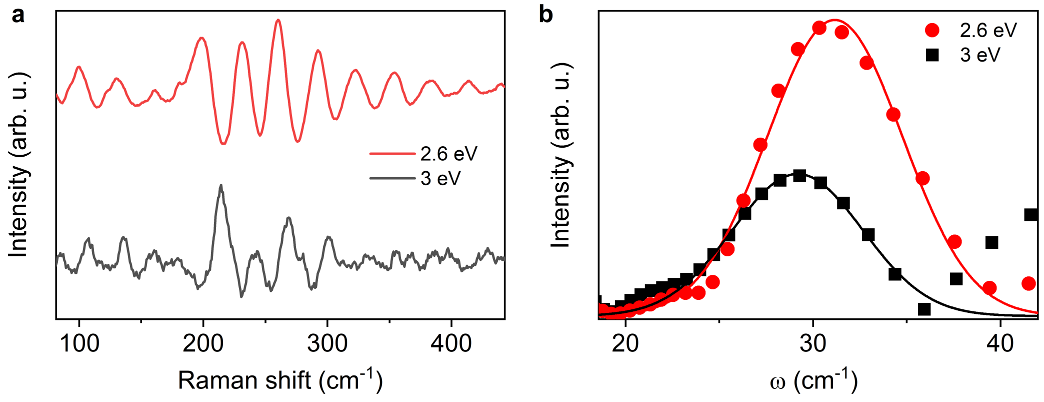

FIG. S7. **HOT PHOTOLUMINESCENCE FOR 2.54 eV EXCITATION FOR $(PEA)_2PbI_4$**

a The evolution of Raman scattering response in function of temperature for 2.54 eV (488 nm) excitation. Arrow indicates the temperature-induced shift of the hot PL. **b** The subtraction of the hot PL signal (blue curve) reveals the characteristic polaron vibronic progression. The red curve (background) was generated by a Savitzky-Golay filter with a 60 point smoothing window.

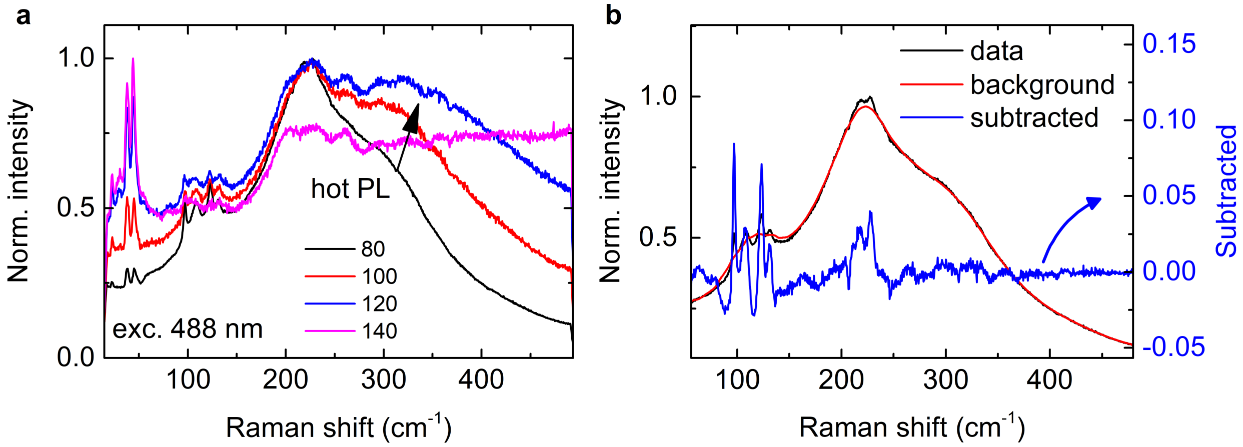

FIG. S8. **ENERGY OF STOKES AND ANTI-STOKES SIGNALS IN VIBRONIC PROGRESSION OF $(PEA)_2PbI_4$**

Extension of the Fig. 4e of the main text, including the signals of the anti-Stokes portion of the spectrum measured with 2.54 and 2.6 eV excitations (see Fig. 3a of the main text). The dashed line is a global linear fit yielding $\omega_1 = 32.1 \pm 0.2\,\mathrm{cm}^{-1}$.

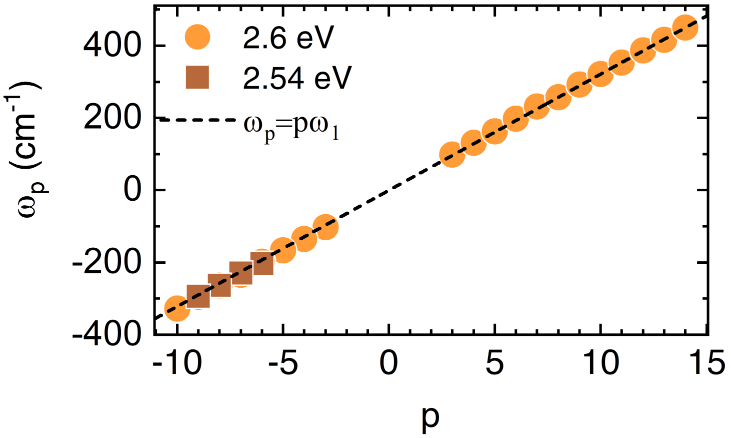

FIG. S9. **FITTING OF ABSORBANCE DATA**

The 1s exciton in both panels is shifted to $0\,\mathrm{cm}^{-1}$. **a** The $(PEA)_2PbI_4$ absorbance data fitted with four main contributions and a broad background (dashed line). **b** The absorbance data for $(BA)_2PbI_4$ calculated on the basis of reflectivity spectrum by means of the Kramers-Kronig transformation[3]. Four main absorbance contributions are present. The negative frequency contribution (red curve) corresponds to the photoluminescence response entering the reflectivity data. For reference the photoluminescence response is presented by dashed line.

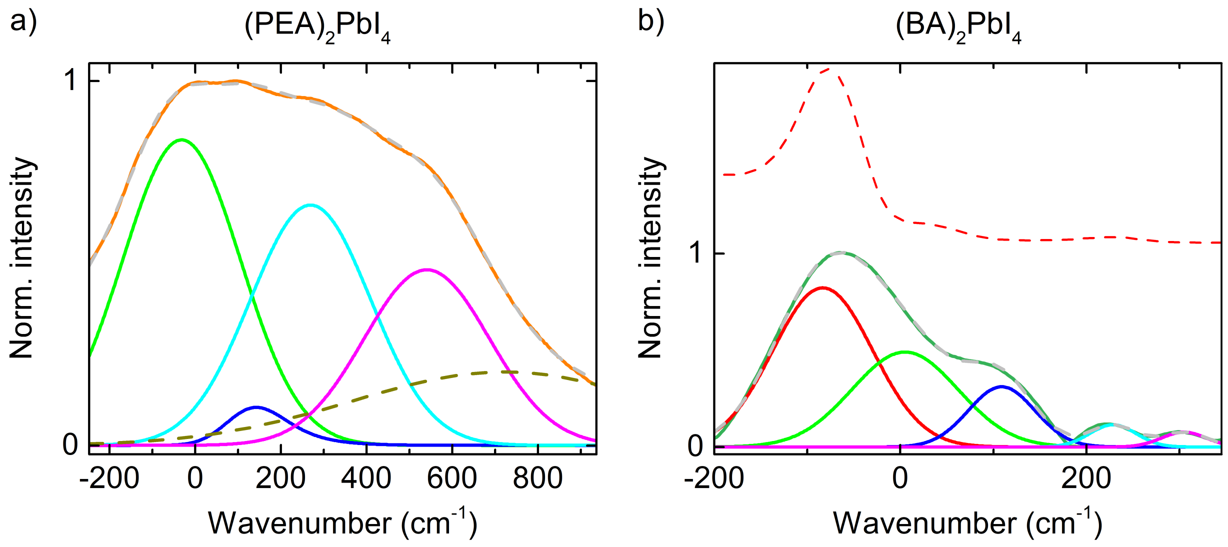

FIG. S10. **COMPARISON OF ABSORBANCE AND RESONANT RAMAN SCATTERING DATA**

a The absorbance (solid black) for $(PEA)_2PbI_4$. The 1s exciton energy is centered at 0 cm^{-1}. Black arrow indicate an approximate energy of optical transitions. A shaded curve is a representative resonant Raman spectrum. The dashed red curve serves as an envelope of high-frequency Raman response. The orange arrows interconnect the respective features in both absorption and Raman scattering curves. A respective data for $(BA)_2PbI_4$ is presented in panel **b**. Note the energy of respective absorption signals is correlated with signals observed in Raman scattering.

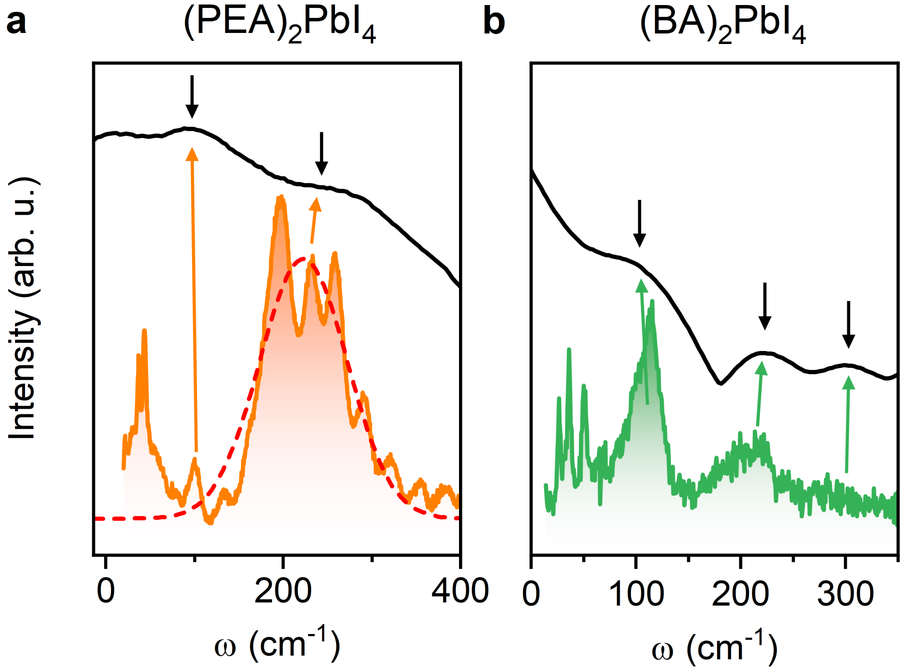

FIG. S11. **INTERMEDIATE ENERGY OVERTONES**

Comparison of resonant Raman spectra for $(BA)_2PbI_4$ and $(PEA)_2PbI_4$. We distinguish two regions of interest (i) intermediate frequency range $50 < \omega < 150$ cm^{-1} and (ii) high-frequency region > 200 cm^{-1}. For both samples a response is observed in both regions, however, the BA-based variant shows much stronger relative response in the range (i) whereas the PEA-based sample shows much stronger response in (ii). For both samples the signal in (i) ($\sim 100\,\mathrm{cm}^{-1}$) is replicated (overtone) at around $\sim$200 cm^{-1}, which is indicated by arrows.

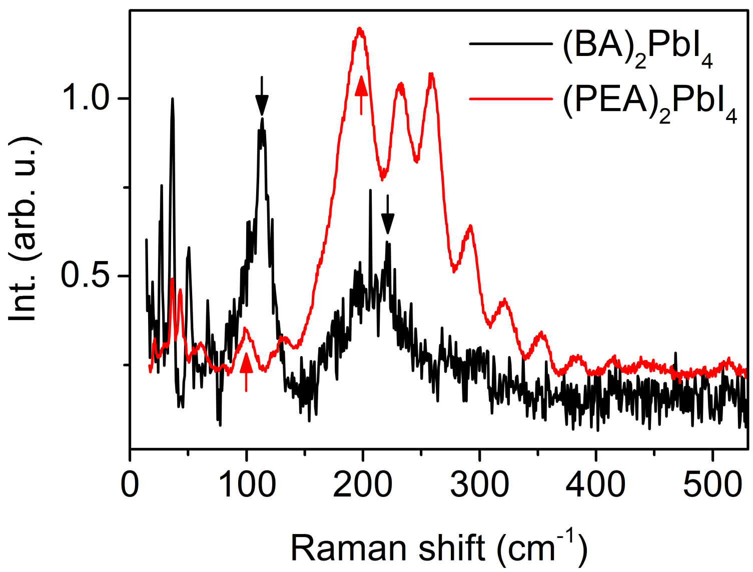

FIG. S12. **POLARIZATION-RESOLVED RAMAN SCATTERING AT DIFFERENT TEMPERATURES**

The evolution of the resonant Raman scattering data with increasing sample temperature for both XX and XY polarizations. The contrast between both polarizations is present in the whole range of measurement temperatures.

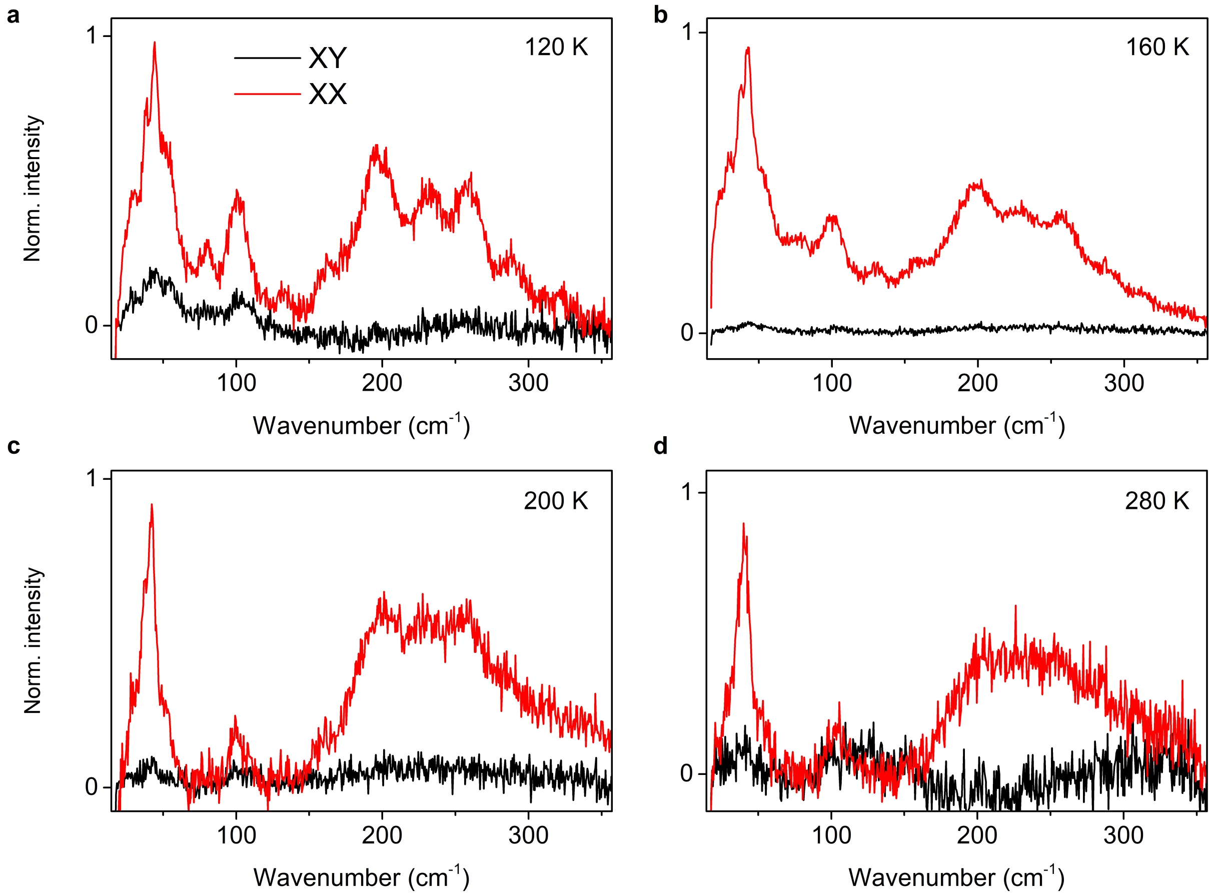

SUPPLEMENTARY TABLE

TABLE S1. **FITTING PARAMETERS.** The parameters used to model the polaronic progression with eq. 1 (see main text). From left: temperature, bandgap, excitation energy, broadening parameter.

	T (K)	E_g (eV)	$\hbar\omega_L$ (eV)	γ (meV)
$(PEA)_2PbI_4$	80	2.604 [2]	2.603	12
$(PEA)_2SnI_4$	80	2.084 [2]	2.329	12
$(BA)_2PbI_4$	80	2.824 [4]	3.002	10

* mateusz.dyksik@pwr.edu.pl

[1] S. Kahmann, H. Duim, H. Fang, M. Dyksik, S. Adjokatse, M. Rivera Medina, M. Pitaro, P. Plochocka, and M. A. Loi, Photophysics of Two-Dimensional Perovskites—Learning from Metal Halide Substitution, Advanced Functional Materials **31**, 2103778 (2021), arXiv:2104.10393.

[2] M. Dyksik, H. Duim, X. Zhu, Z. Yang, M. Gen, Y. Kohama, S. Adjokatse, D. K. Maude, M. A. Loi, D. A. Egger, *et al.*, Broad tunability of carrier effective masses in two-dimensional halide perovskites, ACS Energy Letters **5**, 3609 (2020).

[3] M. Dyksik, M. Baranowski, A. Leblanc, A. Surrente, M. Karpińska, J. M. Urban, Ł. Kłopotowski, D. K. Maude, N. Mercier, and P. Plochocka, Influence of oversized cations on electronic dimensionality of d-MAPbI 3 crystals, Journal of Materials Chemistry C **8**, 7928 (2020).

[4] S. Neutzner, F. Thouin, D. Cortecchia, A. Petrozza, C. Silva, and A. R. Srimath Kandada, Exciton-polaron spectral structures in two-dimensional hybrid lead-halide perovskites, Physical Review Materials **2**, 064605 (2018), arXiv:1803.02455.
